# miR-375 Mediated Acquired Chemo-Resistance in Cervical Cancer by Facilitating EMT

**DOI:** 10.1371/journal.pone.0109299

**Published:** 2014-10-16

**Authors:** Yuanming Shen, Jiansong Zhou, Yang Li, Feng Ye, Xiaoyun Wan, Weiguo Lu, Xing Xie, Xiaodong Cheng

**Affiliations:** Department of Gynecologic Oncology, Women’s Hospital, School of Medicine, Zhejiang University, Hangzhou, China; National Institute of Health - National Cancer Institute, United States of America

## Abstract

Acquired chemo-resistance is one of the key causal factors in cancer death. Emerging evidences suggest that miRNA and epithelial–mesenchymal transition play critical roles in the chemo-resistance in cancers. Here, we showed the association of paclitaxel-resistance with miR-375 over-expression and epithelial–mesenchymal transition inducement in cervical cancer. Using different cervical cancer cell models, we found that paclitaxel transiently induced up-regulation of miR-375 expression, proliferation inhibition, transition from epithelial to mesenchymal phenotype, and consequently impaired paclitaxel sensitivity. Forced over-expression of miR-375 may suppress Ecadherin expression by a directly targeting pathway, which led to paclitaxel resistance. Contrarily, re-expression of Ecadherin partly reversed epithelial–mesenchymal transition phenotype and miR-375 induced paclitaxel-resistance. Our findings suggest that paclitaxel-induced miR-375 over-expression facilitates epithelial–mesenchymal transition process via directly targeting Ecadherin, proliferation inhibition, and consequently results in chemo-resistance in cervical cancer cells. A reversion of miR-375 or Ecadherin expression may be a novel therapeutic approach for overcoming chemo-resistance in cervical cancer.

## Introduction

Emerging evidences have revealed that chemo-resistance is associated with epithelial mesenchymal transition (EMT) in cancer cells, and the acquisition of chemo-resistance in cancer cells is accompanied by a transition from an epithelial to a mesenchymal phenotype [1]–[3]. For instance, various drug-resistant cancers, including oxaliplatin-resistant colorectal cancer cells [4], paclitaxel-resistant ovarian cancer cells [5], and gefitinib-resistant lung cancer cells [6], present the transition from epithelial to mesenchymal phenotype along with a loss of epithelial adhesion molecule Ecadherin and a gain of mesenchymal markers such as vimentin, Ncadherin, and fibronectin. More recent studies also have shown that chemotherapy agents can facilitate the epithelial to a mesenchymal phenotypic transformation in residual surviving cancer cells which may be one of the critical steps in acquired drug resistance in cancers [7], [8]. Thus, acquirement of EMT becomes a key process contributing to the malignant phenotypes of cancer cells in resistance to chemotherapy. Therefore, the intervention of EMT process has been proposed as a therapeutic approach against acquired chemo-resistance.

MicroRNAs (miRNAs) act as important gene regulators in human genomes and their aberrant expression may promote not only tumori-genesis and tumor aggressiveness but also resistance to chemotherapy [9]. More recently, the studies have revealed that miRNAs play an integral role in modulating EMT, such as miR-200s that regulates EMT through inhibiting ZEB1/2, a transcription repressor of Ecadherin [1], [10], [11]. We recently reported that the expression of miR-375 was reduced in cervical cancer cells [12] and enforced over-expression of miR-375 significantly inhibited proliferation and blocked G1-S cell cycle transition [13]. In another study, we further observed that miR-375 was up-regulated in paclitaxel-treated cervical cells and tissues, and forced over-expression of miR-375 decreased paclitaxel sensitivity in cervical cancer [14]. However, the precise molecular mechanism underling miR-375 regulated drug resistance remains to be explored. By the miRNA targets gene prediction, we found a binding site between miR-375 and Ecadherin 3′-UTR, which indicated that Ecadherin was a potential direct target of miR-375. Therefore, we assume that miR-375 may participate in EMT regulation and acquired paclitaxel-resistance in cervical cancer cells.

In the present study, we found for the first time that paclitaxel inhibits proliferation, induces EMT, and up-regulates miR-375 expression simultaneously in cervical cancer cells. And miR-375 over-expression directly inhibited Ecadherin expression and facilitated paclitaxel-resistance. Thus, there is a distinct positive feedback mechanism among paclitaxel, miR-375, and EMT that leads to acquired chemo-resistance phenotypes in cervical cancer cells. Such a regulatory feedback loop results in miR-375 over-expression, the epithelial to a mesenchymal phenotypic transformation, and consequently acquirement of paclitaxel-resistance. Anyway, it could be believed that a break of such a feedback loop would, at least partly, overcome chemo -resistance in cervical cancer.

## Materials and Methods

### Patients and samples

The samples of cancer tissue were collected from 23 cervical squamous cell carcinoma patients with FIGO (2009) stage IB_2_ or IIA_2_ who underwent neo-adjuvant chemotherapy followed by type III radical hysterectomy between November 1, 2008 and May 30, 2010 in Women’s Hospital, School of Medicine, Zhejiang University. The collection of all samples was approved by the Ethical Committee for Clinical Research from the Women’s Hospital, School of Medicine, Zhejiang University and written informed consents were obtained.

All patients underwent 2 cycles of intravenous neo-adjuvant chemotherapy (paclitaxel 135 mg/m^2^ and cisplatin 75 mg/m^2^, 3-week interval) before surgery. The effect of chemotherapy was evaluated according to the Response Evaluation Criteria in Solid Tumors (RECIST) [15] as previously described^14^. Of all 23 patients, mean age was 36.5 years (range 20 to 65 years), had pre- and post-chemotherapy cervical tumor samples. The specified composition of each samples were determined by an experienced pathologist.

### Cell culture and EMT inducement

Two human cervical cancer cell lines, SiHa and CaSki were cultured as previously described^14^. Human recombinant TGF-β1 and EGF-β were obtained from ProSpec-Tany Techno Gene Ltd (Israel), respectively used at a final concentration of 5 ng/ml and 2 ng/ml. The cancer cell lines were seeded 1×10^5^/well in 6-well plates and incubated in serum-free medium overnight, and then treated with TGF-β1 or EGF-β for 72 h respectively, to induce the EMT.

### RNA Extraction and Real-Time RT-PCR

Total RNAs containing miRNAs were extracted from liquid nitrogen preserved tissues or the harvested cells using 1 ml TRIZOL reagent (Invitrogen, Carlsbad, CA, USA) following the manufacturer’s instructions. cDNA was synthesized with the PrimeScript RT reagent Kit (TaKaRa Otsu, Shiga, Japan). Quantitative RT-PCR (qRT-PCR) for miRNA and mRNA was performed as described previously^14^. The sequences of all primers are given in Table 1.

**Table 1:** Sequences of primers for miRNAs, RNU6, and EEF1A1.

miR-375 RT	5′-GTCGTATCCAGTGCAGGGTCCGAGGTATTCGCACTGGATACGACTCACGC-3′
miR-375 forward	5′-AGCCGTTTGTTCGTTCGGCT-3′
miR-375 reverse	5′-GTGCAGGGTCCGAGGT-3′
U6 snRNA RT	5′-AACGCTTCACGAATTTGCGT-3′
U6 snRNA forward	5′-CTCGCTTCGGCAGCACA-3′
U6 snRNA reverse	5′-AACGCTTCACGAATTTGCGT-3′
EEF1A1 forward	5′-TGCGGTGGGTGTCATCAAA-3′
EEF1A1 reverse	5′-AAGAGTGGGGTGGCAGGTATTG-3′

### Cell Proliferation Assay and MTS Assay

To determine the effect of miR-375 and paclitaxel on proliferation of cervical cell lines, the cell proliferation was determined with MTT assay and CellTrace CFSE cell proliferation assay at 24, 48, 72, 96 hours and 5 days respectively. For viability experiments, cervical cancer cells with or without 10 nM paclitaxel treatment 72 hours were plated (SiHa, 3×10^3^/well in 96-well plate and 2×10^5^/well in 6-well plate; CaSki, 2×10^3^/well in 96-well plate and 1×10^5^/well in 6-well plate) and cultured overnight. In MTT assay the absorbance of samples was measured with a spectrophotometer reader at 490 nm. CellTrace CFSE cell proliferation assay was performed by staining activated cervical cancer with CellTrace CFSE cell proliferation dye (2.5 µM) (Invitrogen) and analyze using by flow cytometry with 488 nm excitation and emission filters. An in vitro paclitaxel chemo-sensitivity of cervical cancer cells was evaluated using MTS assay (Promega, Madison, WI) as previously described [12]. Four replicate wells were used for each analysis, and at least three independent experiments were performed.

### Western Blot Analysis

A total of 50 mg protein was separated by denaturing 8–15% SDS–polyacrylamide gel electrophoresis. Western analysis was conducted as previously described [12]. The films were analyzed by densitometry with a VersaDoc Imaging System; Model 3000 (BioRad) using Quantity One software. Analysis of variance with Bonferroni correction for multiple tests was used to determine significance. The primary antibodies used were anti-Ncadherin (1∶5000), anti-Ecadherin (1∶2000), anti-fibronectin (1∶1000), anti-vimentin (1∶1000) and anti-actin (1∶2000) as an endogenous control, all from Epitomics Biotechnology (Epitomics).

### Immunohistochemistry

Sections (6 µm thick) of tumor tissue were cut from paraffinised 23 couples of self-paired pre-and post-chemotherapy cervical cancer samples, and immunohistochemistry analysis and scoring were described previously^12^. The primary antibodies used for immunohistochemistry analysis were anti-Ecadherin antibody (1∶1000) and anti-actin antibody as an endogenous control (1∶2000), both from Epitomics Biotechnology (Epitomics).

### Lentivirus construction and transfection

To generate miR-375 and Ecadherin over-expression stable transfectants, cervical cancer cells were transfected with lentiviral expressing vectors. The plasmid construction and lentivirus package were complete in GENECHEM Company. The pre-miR-375 sequences were amplified and cloned into the pGCsil-GFP vector using the following primers: (Forward) hsa-pre-miR-375-XhoI-F, ACCGCTC GAGCCCCGCGACGAGCCCCTCGC; (Reverse) hsa-pre-mi-R-375-MfeI-R, ACCGCAATTGAAAAAGCCTCACGCGAGCCGAACG. The hsa-CDH1 (CDS) gene was amplified and cloned into a pGC-FU-GFP vector using the following primers: (Forward) 5′-GAGGATCCCCGGGTACCGGTCGCCACCATGGGCCCTTGGAGCCGCAG-3′, (Reverse) 5′-TCATCCTTGTAGTCGCTAGCGTCGTCCTCGCCGCCTCCGTACATG-3′. Viruses packaging were performed in HEK 293 T cells after the cotransfection of 20 mg pGCsil-GFP-pre-miR-375 vector or pGC-GFP-CDH1 vector with 15 mg of the packaging plasmid pHelper 1.0 Vector and 10 mg of the envelope plasmid pHelper 2.0 vector using Lipofectamine 2000. Viruses were harvested 48 h after transfection, and viral titers were determined. The titers of the viral used in this study were in the range of 5×10^8^∼2×10^9^ TU/mL.

To generate miR-375 and Ecadherin over-expression stable transfectants, cervical cancer cells were transfected with lentiviral expressing vectors as previously described [12]. An MOI = 5 was used for SiHa and MOI = 10 was used for CaSki. After 4∼7 d of transfection, stable clones were selected with GFP by flow cytometry and harvested for further experimentation. An empty pGC FU-GFP-NC-LV vector was used as a negative control.

### Luciferase Activity Assay

To investigate whether CDH1 expression was regulated by miR-375, a psicheck-2 dual-luciferase miRNA target expression vector (psicheck-2 -UTR vector) was used to perform the luciferase activity assay. The wild and mutant type 3′-UTR of CDH1 containing predicted miR-375 binding site was synthesized and inserted into the 3′-UTR region downstream of the firefly luciferase gene of psicheck-2 vector (psicheck-2 -UTR) using the Xho I and NotI I restriction sites. All constructs were verified by sequencing. After cotransfected with miR-375 mimics (20 uM) and reporter vectors (0.2 µg/mL), luciferase activities were measured at 24 hours using a Dual-Glo luciferase assay system (Promega). Relative luciferase activity in all figures refers to firefly luciferase activities normalized to Renilla luciferase. Values cells with empty psicheck-2 vector were set equally to 1.

### Statistical Analysis

The experiments were repeated at least three times. Results are expressed as mean ± s.e.m. An independent Student’s t-test or an ANOVA was used to compare continuous variables. Pearson’s correlation coefficient test was used to assess the correlation between two continuous variables. P<0.05 was considered statistically significant.

## Results

### Paclitaxel induced EMT and inhibited proliferation simultaneously in cervical cancer cells

**Figure 1:**
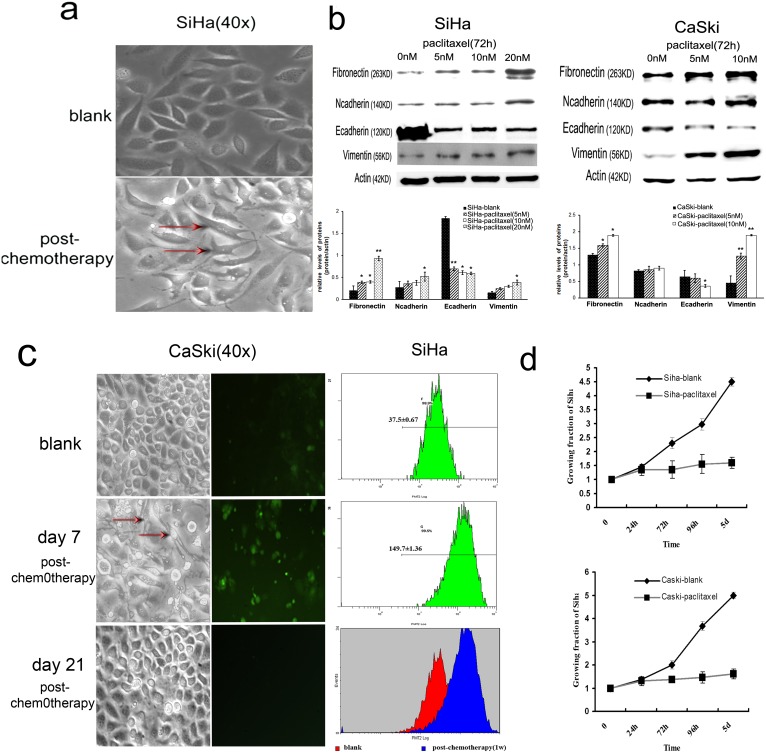
Paclitaxel inhibits proliferation, induces EMT, and up-regulates miR-375 expression simultaneously in cervical cancer cells. (**a**) Visible morphological changes from “cobblestone”-like to “fibroblast”-like cells was observed in SiHa after 72 h paclitaxel treatment. The representative cells with morphological changes were labeled (**b**) Immunoblotting measured the expression of Ecadherin and other EMT related proteins (vimentin, Ncadherin, and fibronectin) in cervical cancer cells (SiHa and CaSki) under different amounts (0, 5, 10, 20 nM) of paclitaxel treatment. The expression of Ecadherin protein was decreased and the expressions of vimentin, Ncadherin, and fibronectin protein were increased in cells treated with paclitaxel for 72 hours in a dose-responsive manner. All the expressions of the proteins were normalized to ß-actin and the data in bar graphs are presented as mean of triple samples from three independent experiments. All error bars indicated s.e.m. (**p≤0.01, *p≤0.05). (**c,** **d**) The transient cell proliferation inhibition and morphological changes induced by paclitaxel were observed. The proliferation changes in SiHa and CaSki cells after paclitaxel administration were analyzed by CellTrace CFSE cell proliferation (**c**) and MTT assay (**d**) at 24 h, 72 h, 96 h, day 5, day 7, day 14, and day 21, respectively. The proliferation rate of paclitaxel treated SiHa and CaSki cells was significantly reduced at day 7 and restored at day 21 after paclitaxel administration. The survived CaSki cells were restored into normal morphology at day 21 after paclitaxel administration.

When the morphology of routinely cultured cervical cancer cells treated with or without paclitaxel were examined by microscopy, we observed that SiHa cell grew as tightly-packed “cobblestone”-like epithelial cells, whereas cells treated with paclitaxel seemed a spindle-like shape flattened and lost the majority of their cell-cell contacts. (Figure 1a) Meanwhile, the expression of Ecadherin protein was decreased (10.5%∼66.6% and 43.75%∼67.6% under 5 nM to 20 nM paclitaxel treatment respectively) and the expressions of Ncadherin and fibronectin protein were increased in a dose-responsive manner. (Figure 1b) Similar effects were also observed in CaSki cells. (Figure 1b, Figure S1).

Further, a transient cell proliferation inhibition induced by paclitaxel was observed by flow cytometry and MTT. SiHa and CaSki cells were routinely cultured with 10 nM paclitaxel, after 72 hours, the paclitaxel were removed and the cells were cultured with complete medium continuously, subsequently the proliferation changes in SiHa and CaSki cells before and after paclitaxel administration revealed at 24 h, 72 h, 96 h, day 5, day 7, day 14, and day 21, respectively. As showed in Figure 1c, 1d, the proliferation rate of paclitaxel treated SiHa and CaSki cells at day 7 was significantly reduced (SiHa, p = 0.002 and p = 2.3×10^−5^; CaSki, p = 3.1×10^−4^ and p = 1.2×10^−5^ respectively), compared with that of blank control, and restored to the levels before drug treatment at day 21. At the same time, the “cobblestone”-like morphological changes induced by paclitaxel in survived cells were also restored to normal morphology at day 21. These facts indicate that paclitaxel transiently inhibits proliferation and induces EMT simultaneously in cervical cancer cells, but all these paclitaxel-induced changes reversed after drug withdrawal.

### Paclitaxel up-regulates miR-375 during inducing EMT and proliferation inhibition

We had explored miR-375 expression changing involved in paclitaxel induced EMT and proliferation inhibition in cervical cancer cells, and the clear dose dependent effect of paclitaxel on miR-375 over-expression was observed^14^. Moreover, the progressive up-regulated expression of miR-375 reached a peak at day 7 after paclitaxel administration, gradually declined after paclitaxel removed and restored to the level before drug treatment at about day 21^14^. In addition, there was a strong negative correlation between the expression of miR-375 and the relative proliferation proportion of cervical cancer cells (SiHa, r = −0.472, P = 1.52×10^−3^; CaSki, r = −0.835, P = 3.03×10^−4^). (Figure S2) Our data revealed that paclitaxel up-regulated miR-375 expression and inhibited proliferation simultaneously in cervical cancer cells but both paclitexel-induced changes reversed after drug withdrawal, suggesting that some of cervical cancer cells survive in paclitaxel, probably though up-regulating miR-375 expression and acquiring malignant mesenchymal phenotypes, then developing a potential to decrease proliferation and resist drug toxicity. These correlation suggest that miR-375 over-expression might have a causal role in chemotherapy induced EMT.

### MiR-375 mediates EMT by directly targeting Ecadherin

**Figure 2:**
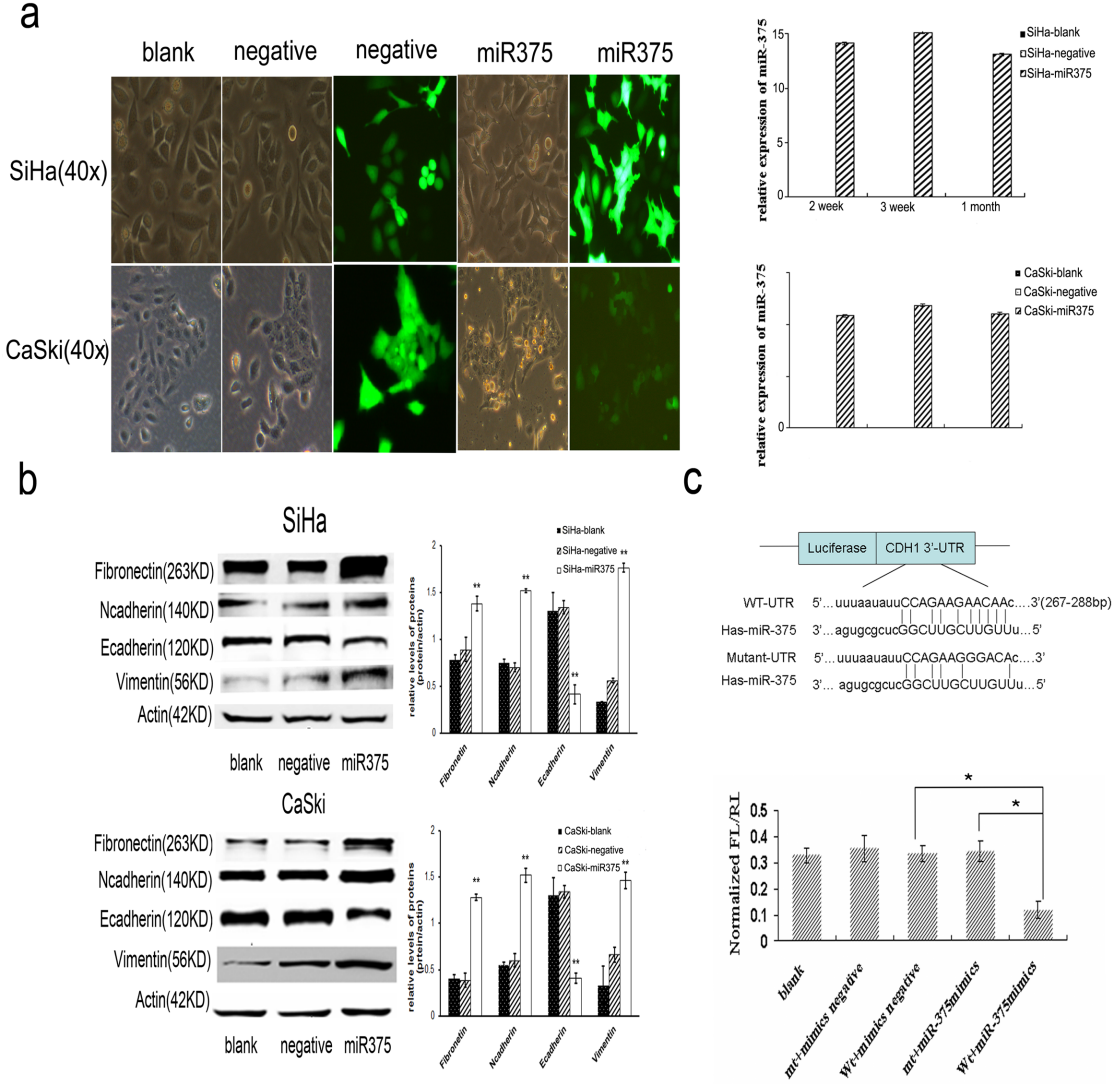
Conditionally over-expressed miR-375 induces EMT via targeting Ecadherin in cervical cancer cells. (a) Phase contrast images of cervical cancer cells infected with the pre-miR-375 or negative lentivirus vector and blank control. (b) Immunoblotting measurement of Ecadherin and EMT related proteins in cervical cancer tercells infected with the pre-miR-375 or negative lentivirus vector. (c) A predicted duplex formation between human CDH1 (Ecadherin) 3′-UTR and miR-375. A reduced luciferase activity was observed after cotransfection of Ecadherin 3′-UTR wild type vector with miR-375, but not mutated or empty vector (p = 0.014). Data in a–c were presented as the mean of triplets samples from independent experiments. All error bars indicated s.e.m. (**p≤0.01, *p≤0.05).

By miRDB and TargetScan search programs analysis, we found a predicted binding of miR-375 with CDH1 (Ecadherin) 3′-UTR, which indicated that Ecadherin was a potential direct target of miR-375 (Figure 2c). To further ascertain that Ecadherin is a target gene of miR-375, we firstly analyze the relationship between Ecadherin and miR-375 expression in cervical cancer cells under different amounts of paclitaxel treatment. As showed in Figure 1b and Figure S2, miR-375 expression in cells was markedly unregulated in a dose dependent manner during paclitaxel-induced EMT process, as well as Ecadherin protein and miR-375 expression were reversely correlated (r = −0.752, P = 2.32×10^−4^). Then we further used the Luciferase Activity Assay to confirm the binding of miR-375 to the 3′-UTR of Ecadherin. (Figure 2c).

To test whether the up-regulation of miR-375 contributed to paclitaxel -induced EMT, the effects of miR-375 over-expression on these events were evaluated. The expression of miR-375 was analyzed by Real-time PCR at the second week, third week and first month after pre-miRNA lentiviral stable transfection. As shown in Figure 2a and 2b, the forced over-expression of miR-375 induced EMT process in cervical cancer cells, characterized by acquisition of fibroblast-like cell morphology, suppression of Ecadherin expression (65%∼69.2%), and enhancement of vimentin, Ncadherin, and fibronectin expression, similar to the effects observed in the paclitaxel treated cells.

Thus, our findings indicate that miR-375 over-expression promotes paclitaxel-induced EMT partly by directly targeting Ecadherin in cervical cancer cells.

### EMT regulates the paclitaxel sensitivity but not miR-375 expression

**Figure 3:**
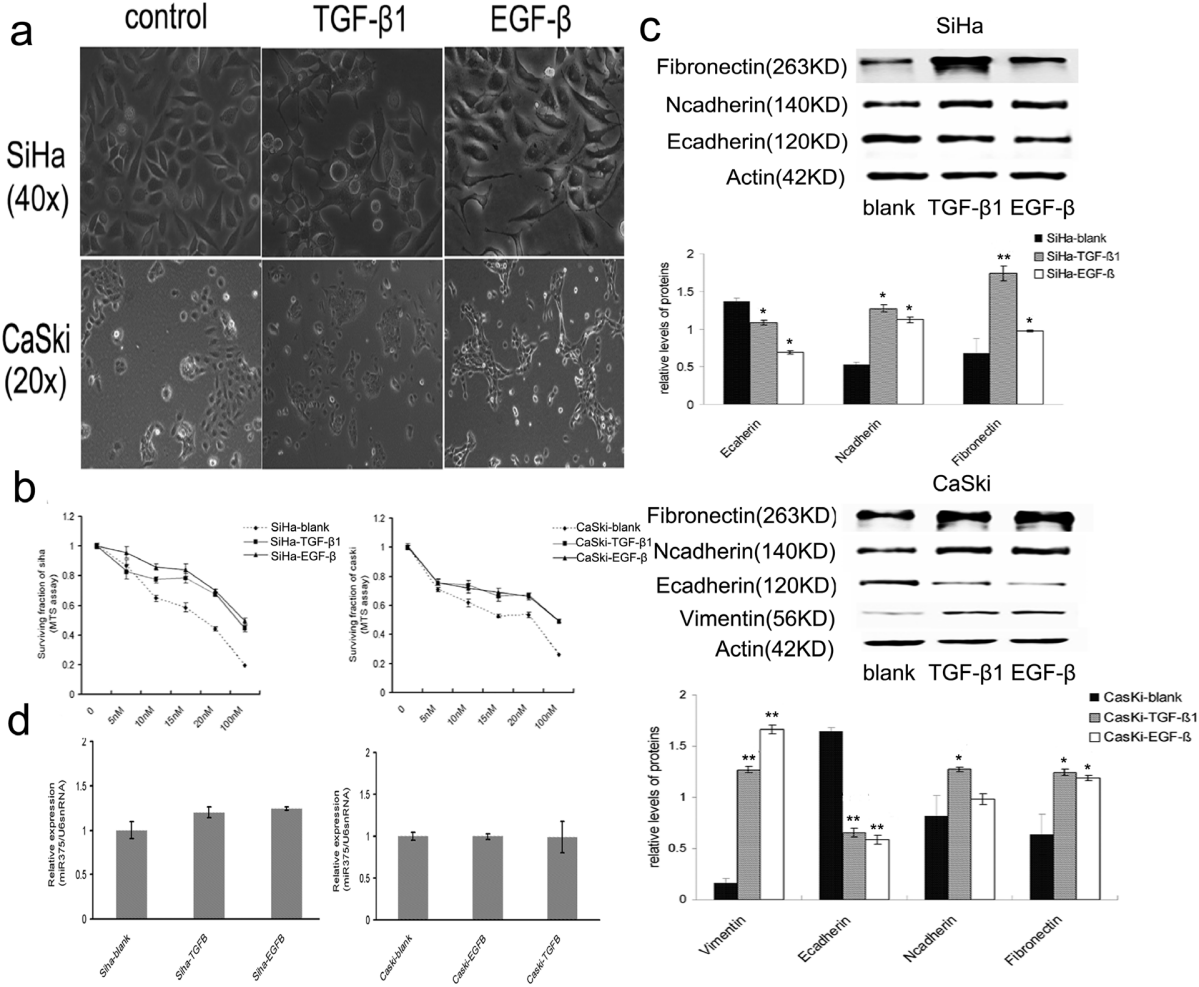
TGF-β1 or EGF-β induces EMT and paclitaxel resistance in cervical cells. (a) Visible morphological changes in SiHa and CaSki under TGF-β1 or EGF-β inducement. (b) MTS analysis of paclitaxel-sensitivity in cervical cancer cells. (c) Immunoblotting analysis of Ecadherin and other EMT related proteins after TGF-β1 or EGF-β induction. (d) miR-375 expression in SiHa and CaSki under TGF-β1 or EGF-β inducement. Data in b–d were presented as mean of triplets samples from independent experiments. All error bars indicated s.e.m. (**p≤0.01, *p≤0.05).

To determine whether EMT may regulate miR-375 expression inversely, the next set of experiments was carried out. We induced EMT by TGF-β1 or EGF-β in cervical cancer cells (SiHa and CaSki) and observed the influence of EMT on paclitaxel sensitivity and miR-375 expression. Stimulated by TGF-β1 or EGF-β, cervical cancer cells underwent EMT accompanying visible morphological changes (Figure 3a), such as appearance of a spindle-like shape in cells, and EMT marker alteration, including decreased Ecadherin protein expression (28.5%∼65.8% for TGF-β1 and 47.5%∼67.6% for EGF-β), as well as increased Ncadherin, vimentin, and fibronectin protein expression (Figure 3c). However, the expression of miR-375 was not changed during TGF-β1 or EGF-β induced EMT process in cervical cancer cells (SiHa, *p* = 0.538; CaSki *p* = 0.542) (Figure 3d).

Further, we delineated the drug sensitivity of cells undergoing EMT using MTS assay. The paclitaxel sensitivity was significantly decreased in SiHa and CaSki cells after TGF-β1 or EGF-β stimulation compared with serum-free culture controls (Figure 3b). The IC50 values were increased in SiHa and CaSki with 5 ng/ml TGF-β1 (95.81±2.53 nM and 66.66±2.32 nM respectively, SiHa, *p* = 0.032; CaSki, *p* = 0.023) or 2 ng/ml EGF-β stimulation (96.74±2.47 nM and 94.78±1.72 nM respectively, SiHa, *p* = 0.038; CaSki *p* = 0.028) compared with serum-free culture controls (24.54±1.37 nM, and 23.98±2.62 nM respectively).

Our findings indicate that TGF-β1 or EGF-β induces EMT but dose not alter miR-375 expression in cervical cancer cells, and mesenchymal cell-like cervical cancer cells induced by TGF-β1 or EGF-β are typically paclitaxel resistant. The significantly upregulated expression of miR-375 in paclitaxel treated cervical caner cells is induced by paclitaxel but not EMT.

### Ecadherin over-expression attenuates the ability miR-375 to mediate EMT and paclitaxel-resistance

**Figure 4:**
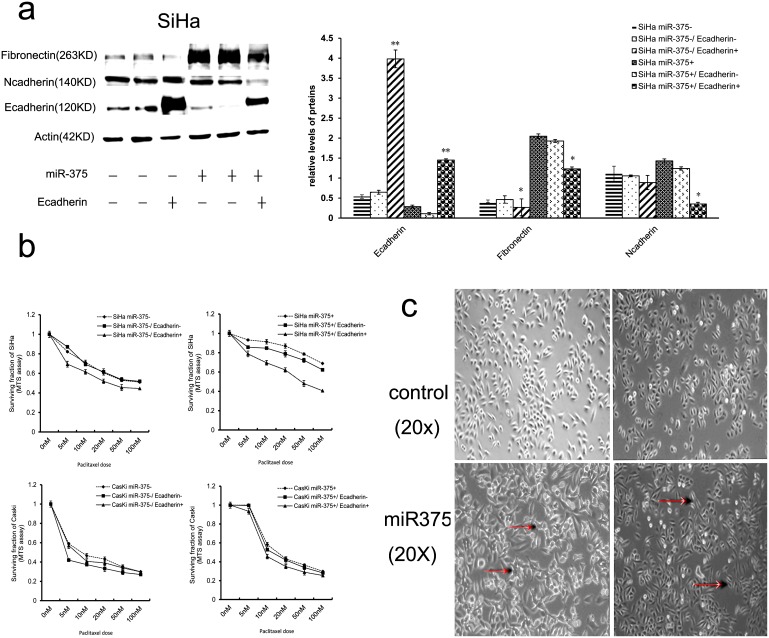
Forced expression of Ecadherin attenuates the ability miR-375 to mediate EMT and paclitaxel-resistance. Immunoblotting (a) and MTS (b) analysis of Ecadherin and other EMT related proteins in SiHa with and without miR-375 overexpression after the Ecadherin-expressing or negative lentivirus vector transfection. Data in (a) and (b) were presented as the mean of triplets samples from independent experiments. All error bars indicated s.e.m. (**p≤0.01, *p≤0∶05). (c) Morphological changes in SiHa with and without miR-375 over-expression after the Ecadherin-expressing or negative lentivirus vector transfection. The representative cells with morphological changes had been labeled.

Ecadherin is not only an important molecule in the initiation of EMT but also a hallmark for EMT and miR-375 mediates EMT by directly targeting Ecadherin.To investigate the role of Ecadherin in EMT and paclitaxel sensitivity and to test whether the up-regulation ofEcadherin can attenuates the effect of miR-375 on EMT and paclitaxel-resistance in cervical cancer cells; the Ecadherin over-expressed lentviral pGC-flag-CDH1 vector was used. We respectively transfected Ecadherin over-expressed lentviral vector and negative control into SiHa and CaSki cells with and without miR-375 over-expression, and observed that the effect of forced over-expression Ecadherin on EMT and paclitaxel-resistance. In Ecadherin over-expressing clones with and without miR-375 over-expression, there was a pronounced and significant increase in the expression of Ecadherin (Figure 4a). In cells without miR-375 over-expression, no remarkable differences of morphology and mesenchymal markers were observed in SiHa and CaSki cells after Ecadherin over-expression, which is probably because their parental cells already exhibited an epithelial phenotype. Contrarily, in cells with miR-375 over-expression, there was a transition from isolated fibroblastic cells to “cobblestone”-like epithelial cells. Meanwhile, the expressions of Ncadherin, vimentin, and fibronectin were significantly decreased after Ecadherin over-expression (Figure 4a, 4c).

The paclitaxel chemo-sensitivity was then evaluated by MTS assays. As expected, paclitaxel sensitivity was significantly increased in Ecadherin lentvirus-transfected cells with and without miR-375 overexpression compared with control cells. The IC50 values were decreased in SiHa and CaSki with Ecadherin overexpression (12.82±2.54 nM and 7.62±0.93 nM respectively, SiHa, *p* = 0.043; CaSki, *p* = 0.038) compared with negative controls (20.73±1.23 nM and 11.68±0.62 nM respectively, SiHa, *p* = 0.028; CaSki, *p* = 0.021). Moreover, the paclitaxel resistance was significantly attenuated after Ecadherin was effectively over-expressed in cervical cells with over-expressed miR-375 compared with negative control (Figure 4b). The IC50 values were decreased in SiHa and CaSki with Ecadherin and miR-375 cotransfection (22.82±3.54 nM and 13.68±0.64 nM respectively, SiHa, *p* = 0.033; CaSki, *p* = 0.038) compared with SiHa and CaSki with negative control and miR-375 cotransfection (82.83±2.23 nM and 20.31±3.01 nM respectively, SiHa, *p* = 0.032; CaSki, *p* = 0.025). Our results revealed that Ecadherin over-expression significantly enhanced paclitaxel sensitivity in cervical cancer cells with or without miR-375 over-expression, suggesting that Ecadherin over-expression dramatically enhances paclitaxel sensitivity, further, re-expression of Ecadherin reverses, at least partly, the EMT phenotype and paclitaxel-resistance mediated by miR-375 in cervical cancer cells.

### miR-375 over-expression correlates with Ecadherin expression in post-chemotherapy human cervical carcinoma tissues

**Figure 5:**
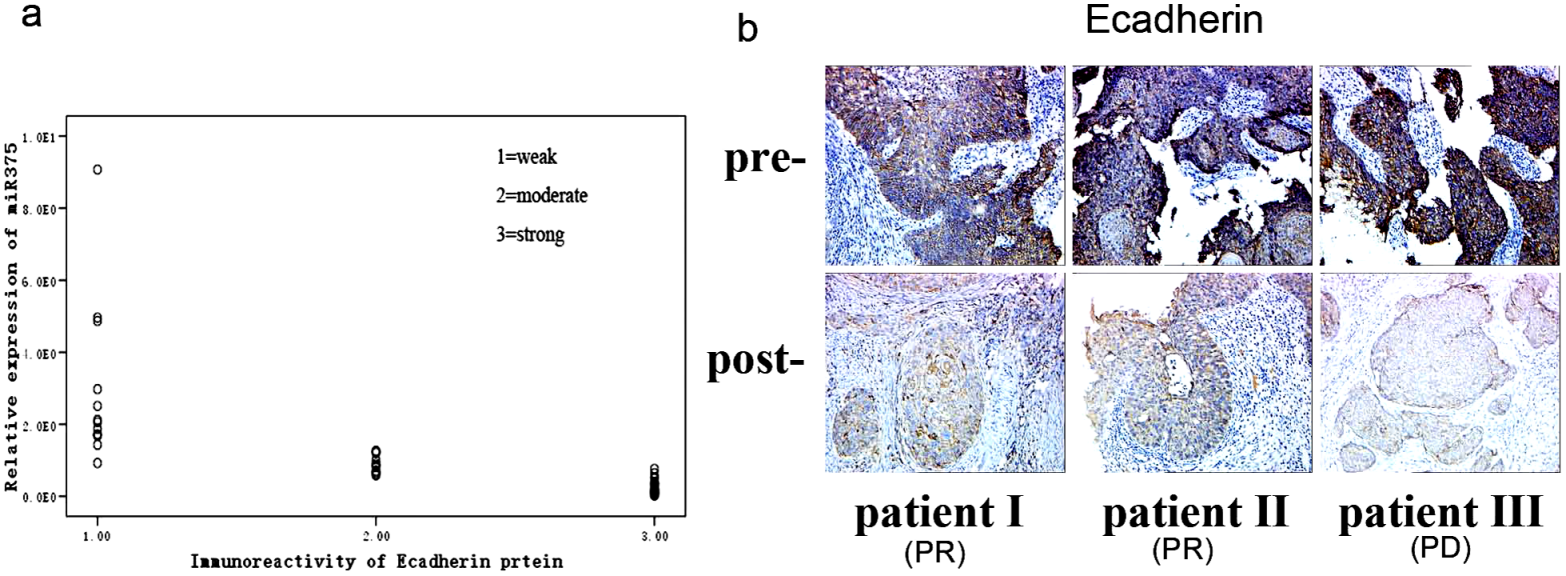
miR-375 over-expression correlates with Ecadherin expression in post-chemotherapy human cervical carcinoma tissues. (a) Ecadherin and miR-375 expression were reversely correlated in all tissues including pre- and post-chemotherapy (r = −0.905, P = 1.81×10^−3^). (b) Staining of human-specific Ecadherin expression in three representatives (1 PD and 2 PR) (×200). A decreased Ecadherin expression was shown in the tissues after chemotherapy (p = 0.0023).

To establish the clinical relevance of miR-375 between Ecadherin expressions under chemotherapy effects, we assessed miR-375 and Ecadherin expression in 23 couples of self paired pre- and post-chemotherapy human cervical cancer tissues. The immunostaining for Ecadherin revealed more extensive and stronger expression in pre-chemotherapy than that in post-chemotherapy tumor tissues (Figure 5). At the same time, miR-375 was markedly upregulated (4.67 fold) in paclitaxel-treated cervical samples, Ecadherin and miR-375 expression were reversely correlated in all tissues including pre- and post-chemotherapy (r = −0.905, P = 1.81×10^−3^) (Table S1).

Of all 23 patients, 19 were chemo-sensitive and 4 chemo-resistance, the upregulated expression of miR-375 is more significantly in chemo-resistance patients when compared post-chemotherapy tissues with their self paired pre-chemotherapy tissues (6.7±2.3 fold in chemo-resistance and 4.35±0.63 fold in chemosensitive patients respectively, *p* = 0.033). But it should be noted that there is no enough power to conclude that miR-375 expression is higher in chemo-resistance patients, partially due to the small sample size for the chemo-resistance patients (N = 4).

## Discussion

Chemo-resistance is one of the key causal factors in cancer relapses and progression. The majority of cancer cells responds well initially to chemotherapy, but eventually develops resistance following drug exposure. Emerging evidences have revealed that cancer cells undergo EMT promotes cell survival and resistance to chemotherapy [3]. Although it has been reported that miRNAs may play an integral role in modulating EMT [16]; however, detailed regulatory procedure remains unexplored. miR-375 was initially found to be expressed in pancreatic islet, where it regulates the secretion of insulin and metabolism of glucose [17]. Recently, miR-375 was identified as an important regulator in tumorgenesis and cancer progression; however, the precise functions of this miRNA remain largely obscure. miR-375 was identified as a tumor suppressor in most of cancers, such as gastric cancer [18], [19], liver cancer [20], head and neck squamous cell carcinoma [21] and prostate cancers [22], but plays an oncogenic role in ERα-positive breast cancer [23], in lung adenocarcinoma patients [24], and in HBV positive HCC patients [25]. Thus, these conflicting reports regarding the association between miR-375 level and cancer suggested a more complex and possibly cancer specific relationship. In another study of ours, we observed the association between miR-375 over-expression and paclitaxel -resistance in cervical cancer^16^. Here, we directly link miR-375 to chemotherapy-induced EMT process in cervical cancer. We found for the first time that paclitaxel inhibits proliferation, induces EMT, and up-regulates miR-375 expression in a dose-dependent manner simultaneously in cervical cancer cells, but all these paclitaxel-induced changes reversed after drug withdrawal. Mechanistically, ectopic over-expression of miR-375 resulted in reduced Ecadherin expression and accompanied by acquired mesenchymal characteristics and chemo-resistance in cervical cancer cells. Thus, our findings suggested that paclitaxel does not eliminate all cervical cancer cells and some of residual cells can survive under drug exposure. During this procedure, those residual cervical cancer cells with up-regulated miR-375 expression probably possess a potential to acquire mesenchymal phenotypes, slow down proliferation, and resist to drug toxicity. Thus, miR-375 induced EMT might play a key role in paclitaxel resistance in cervical cancer cells. Both cell proliferation inhibition and mesenchymal phenotype have been regarded as features of dormant tumor cells that are resistant to chemotherapy [26], [27]. It is possible that residual surviving cells go into the status of tumor dormancy. More importantly, those dormant-like cells escaping from killing by drug can *de novo* down-regulate miR-375 expression and reconvert proliferation after drug withdrawal. Clinically, it is those revival cells that present the progression or recurrence and consequently contribute to poor prognosis of cancer patients. There are only few reports associating miR-375 with chemo-resistance. However, recently a reduced expression of miR-375 was found in tamoxifen resistant cell lines and re-expression of miR-375 reverses both tamoxifen resistance and accompanying EMT-like properties in breast cancer [28]. This conflicting report indicated that the role of miR-375 in regulating drug-sensitivity is possibly cancer and anticancer agents’ specific relationship such as in regulating tumorgenesis and cancer progression.

Previous studies showed that miRNAs regulated EMT process but EMT in turn regulated miRNA expression. For example, miR-200 family members regulate EMT, through targeting and suppressing ZEB, and they are in turn repressed in a timely correlated manner during Snail-induced EMT in MCF7 breast cancer cells [29]. To exclude the possibility that miR-375 was regulated by EMT, we examined miR-375 expression during EMT process induced by transforming growth factor-b (TGF-β) and epidermal growth factor-b (EGF-β) treatment in cervical cancer cells. Both factors are multifunctional cytokines that induce EMT in multiple cell types [30], [31]. Our results showed that miR-375 was regulated only during paclitaxel induced EMT but not TGF-β and EGF induced EMT. Thus, our findings recognize that the upregulated expression of miR-375 in paclitaxel treated cervical caner cells is only induced by paclitaxel but not EMT.

Ecadherin is a calcium dependent cell adhesion molecule which plays an important role in the growth and development of cells via controlling tissue architecture and maintaining tissue integrity [32]. Recently some studies have demonstrated that reduction and/or loss of Ecadherin expression is a hallmark of the EMT process, which is required for enhancing drug resistance of cancer cells [33]. Here, we observed that Ecadherin is a direct target of miR-375 in cervical cancer cells; moreover, re-expression of Ecadherin partly reversed the EMT phenotype and paclitaxel -resistance induced by miR-375 in cervical cancer cells. These data suggest that Ecadherin is a major molecule in regulating chemo-sensitivity.

In summary, our findings for the first time, to our knowledge, suggest that paclitaxel up-regulates miR-375 expression and over-expressed miR-375 induces EMT process via directly targeting Ecadherin, proliferation inhibition, and consequently results in chemo-resistance in cervical cancer cells. A reversion of miR-375 or Ecadherin expression may be a novel therapeutic approach for overcoming chemo-resistance in cervical cancer.

## Supporting Information

Figure S1 **Visible morphological changes in CaSki after chemotherapy.** Visible morphological changes from “cobblestone”-like to “fibroblast”-like cells were observed in CaSki after 72 h paclitaxel treatment.
(TIF)

Figure S2 **The correlation between miR-375 and proliferation.** (a) miR-375 inhibited proliferation in cervical cancer cells. (b) The progressive up-regulated expression of miR-375 reached a peak at day 7 after paclitaxel administration, gradually declined after paclitaxel removed and restored to the level before drug treatment at about day 21. (c) miR-375 expression in cells was markedly unregulated in a dose dependent manner during paclitaxel treatment.
(TIF)

Table S1 **miR-375 over-expression correlates with Ecadherin expression in cervical cancer tissues.** Staining of human-specific Ecadherin expression in paraffin embedded 23 couples of self-paired pre-and post-chemotherapy cervical cancer samples (19 chemo-sensitive, 4 chemo-resistance). Ecadherin and miR-375 expression were reversely correlated (r = −0.905, P = 1.81×10–3).
(DOCX)
