# Supplementary figures and images for: miR-375 Mediated Acquired Chemo-Resistance in Cervical Cancer by Facilitating EMT
Source: PLoS One. 2014 Oct 16;9(10):e109299. doi: 10.1371/journal.pone.0109299 (PMC4199595; doi:10.1371/journal.pone.0109299)

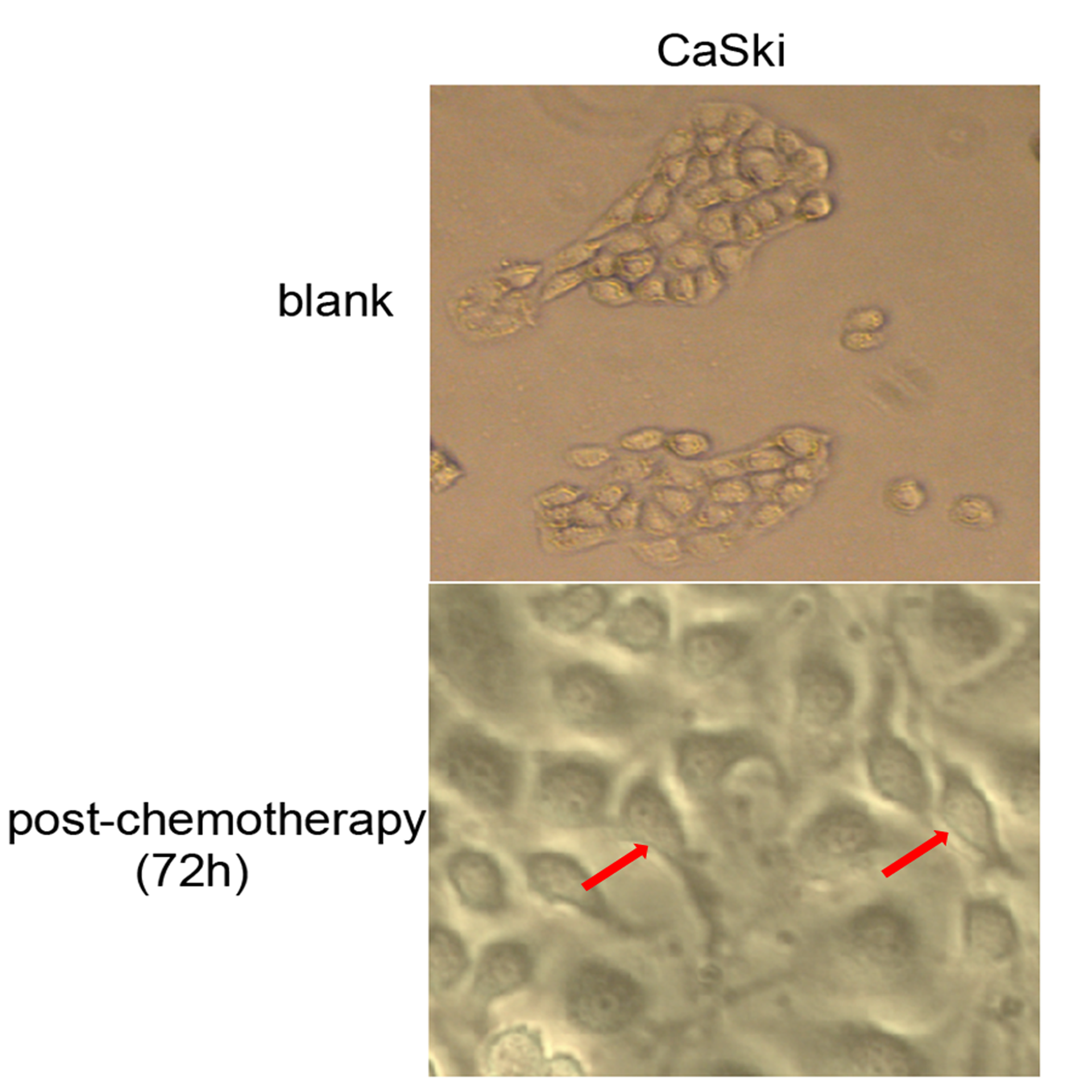

Supplement: Figure S1 — Visible morphological changes in CaSki after chemotherapy. Visible morphological changes from “cobblestone”-like to “fibroblast”-like cells were observed in CaSki after 72 h paclitaxel treatment. (TIF) [file pone.0109299.s001.tif]

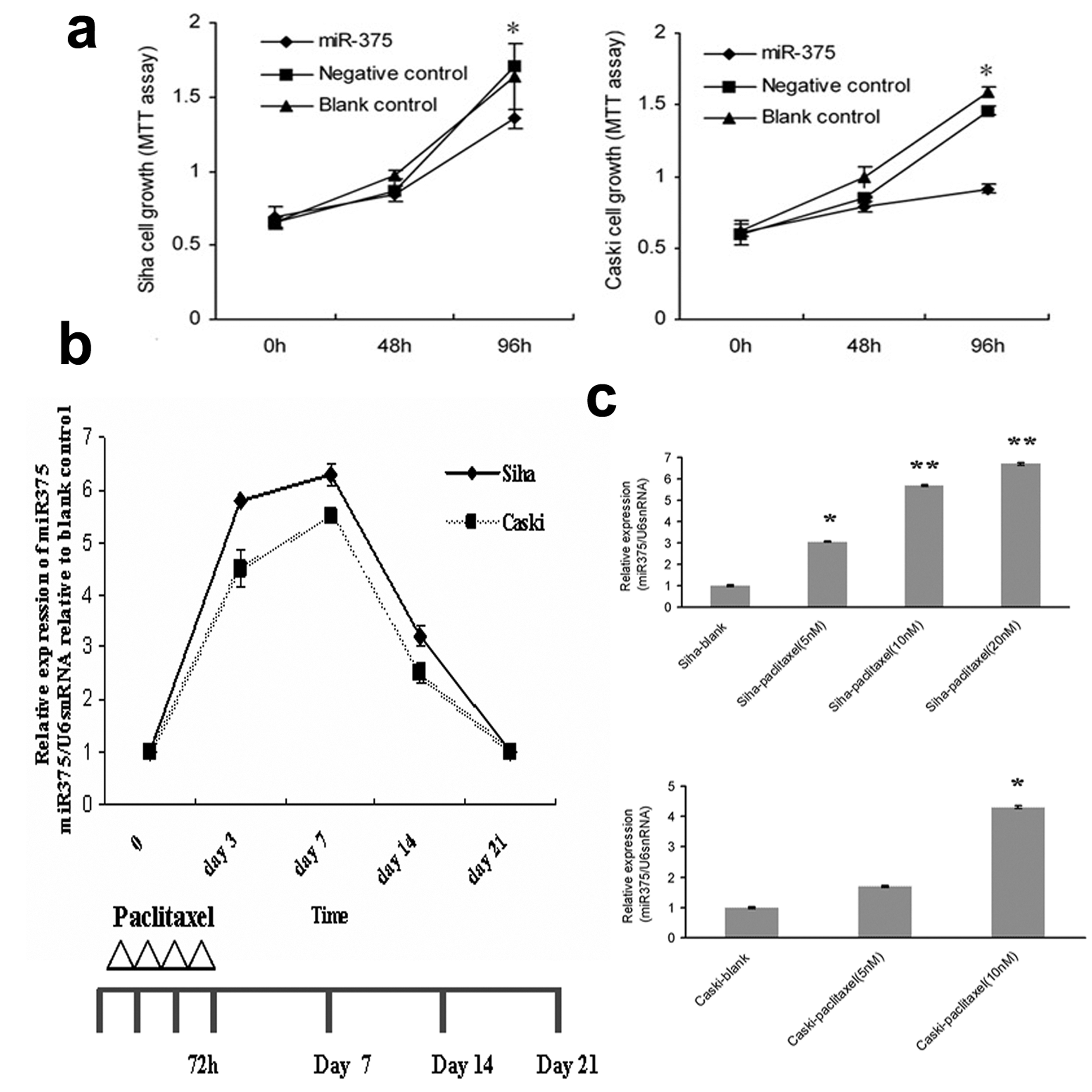

Supplement: Figure S2 — The correlation between miR-375 and proliferation. (a) miR-375 inhibited proliferation in cervical cancer cells. (b) The progressive up-regulated expression of miR-375 reached a peak at day 7 after paclitaxel administration, gradually declined after paclitaxel removed and restored to the level before drug treatment at about day 21. (c) miR-375 expression in cells was markedly unregulated in a dose dependent manner during paclitaxel treatment. (TIF) [file pone.0109299.s002.tif]
