# Supplementary material for: miR-375 Mediated Acquired Chemo-Resistance in Cervical Cancer by Facilitating EMT
Source: PLoS One. 2014 Oct 16;9(10):e109299. doi: 10.1371/journal.pone.0109299 (PMC4199595; doi:10.1371/journal.pone.0109299)
Supplement: Table S1 — miR-375 over-expression correlates with Ecadherin expression in cervical cancer tissues. Staining of human-specific Ecadherin expression in paraffin embedded 23 couples of self-paired pre-and post-chemotherapy cervical cancer samples (19 chemo-sensitive, 4 chemo-resistance). Ecadherin and miR-375 expression were reversely correlated (r = −0.905, P = 1.81×10^–3^). (DOCX) [file pone.0109299.s003.docx]

**TableS1: miR-375 over-expression correlates with Ecadherin expression in cervical cancer tissues**
